# 616. Patient Demographics and Isolate Characteristics for Candida auris Cases — New York City, 2016–2024

**DOI:** 10.1093/ofid/ofaf695.189

**Published:** 2026-01-11

**Authors:** Michelle E Chang, Thomas Portier, Katelynn Devinney, Molly M Kratz, Addie Crawley, Nicole Burton, William Greendyke, Tristan D McPherson

**Affiliations:** Centers for Disease Control and Prevention, New York, NY; NYC Department of Health and Mental Hygiene, Queens, New York; NYC Department of Health and Mental Hygiene, Queens, New York; NYC Department of Health and Mental Hygiene, Queens, New York; NYC Department of Health and Mental Hygiene, Queens, New York; NYC Department of Health and Mental Hygiene, Queens, New York; NYC Department of Health and Mental Hygiene, Queens, New York; New York City Department of Health and Mental Hygiene, New York, NY

## Abstract

**Background:**

*Candida auris* is a drug-resistant fungal pathogen that can colonize skin and cause invasive infections, typically among patients in healthcare settings. Since *C. auris* was first reported in the United States in 2016, more cases have been reported in New York than any other state. Laboratory-confirmed *C. auris* must be reported to the New York City (NYC) Health Department. We characterized the epidemiology of *C. auris* cases in NYC to guide strategies to prevent transmission.

**Figure:**
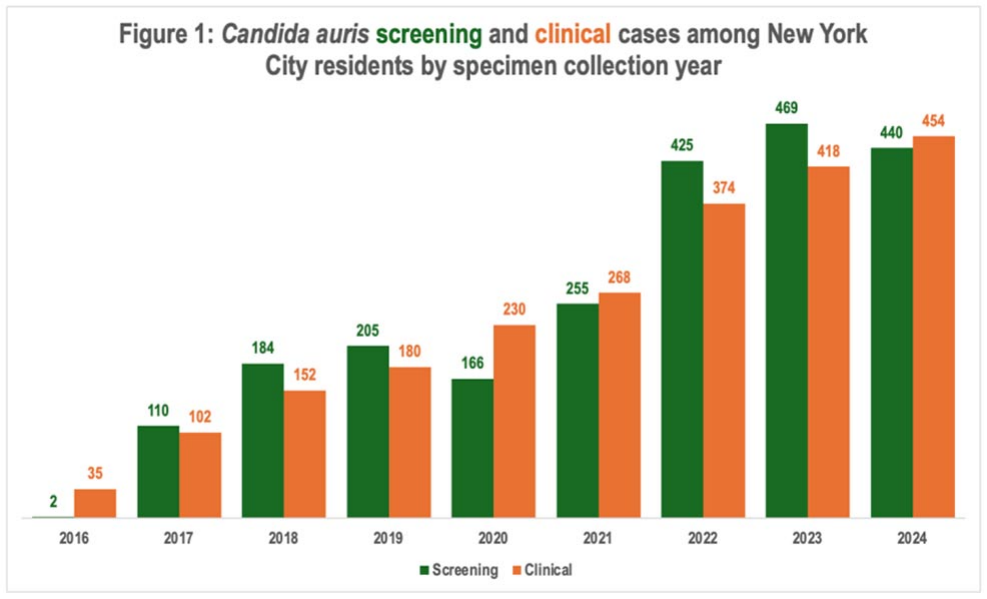

**Figure:**
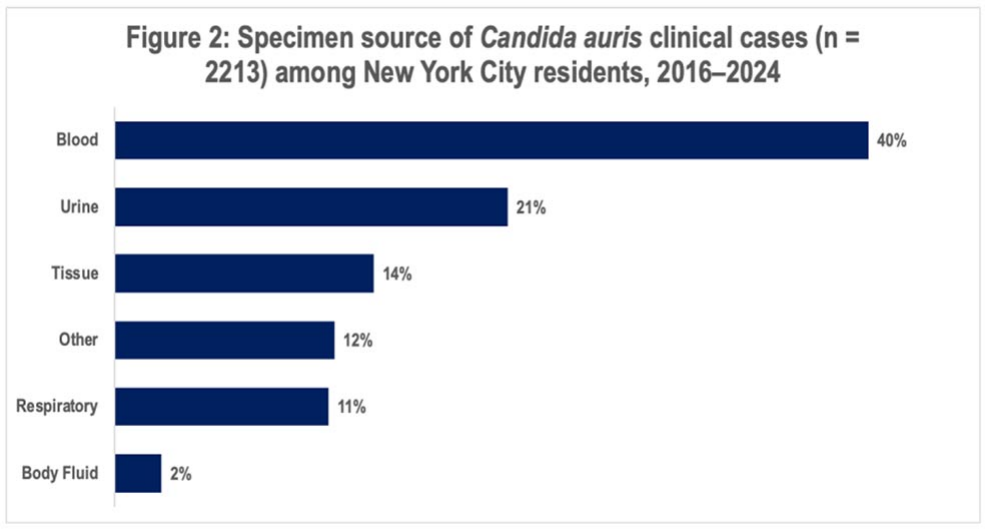

**Methods:**

We examined clinical and public health laboratory-reported *C. auris* specimens to enumerate screening and clinical cases among NYC residents during 2016–2024 using the Council of State and Territorial Epidemiologists’ 2023 case definition. A person colonized or infected with *C. auris* is considered colonized indefinitely and counted as a single case; screening cases can later be categorized as clinical cases. We examined age, sex, facility type, and specimen source among cases over time. We described antifungal susceptibility testing (AFST) results for a subset of isolates during 2019–2024.

**Figure:**
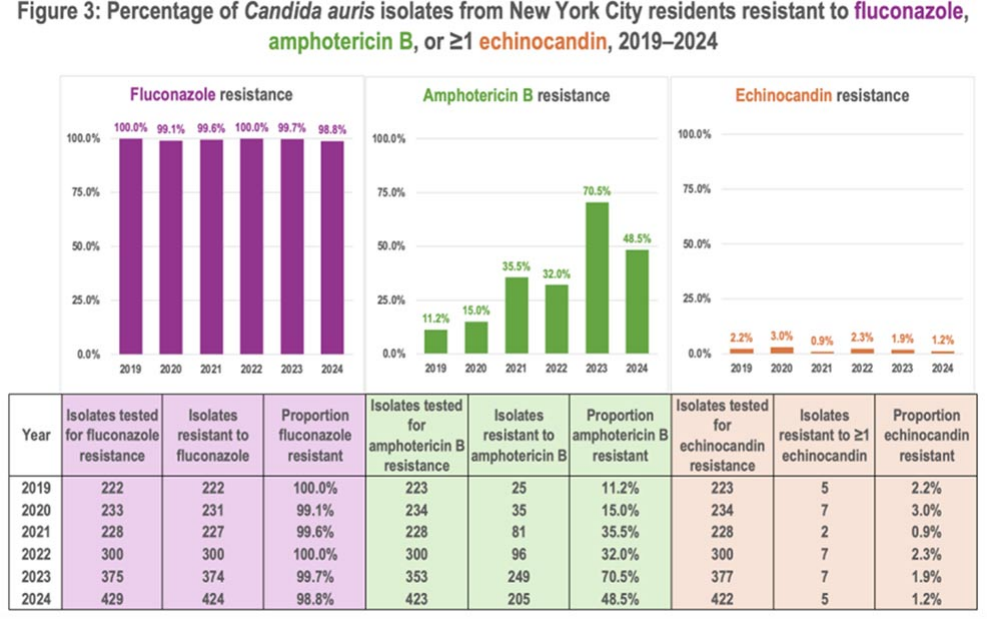

**Results:**

We identified 8,412 specimens representing 2,256 screening and 2,213 clinical cases. Cases increased over time (Figure 1). Among clinical cases, median patient age was 68 years (standard deviation: 16 years), 60% were male, 37% resided in a skilled nursing facility (SNF), and the most common specimen sources were blood (40%) and urine (21%) (Figure 2). Among 7,334 specimens reported during 2019–2024, 1,795 (24%) included AFST results, representing 863 (45%) of 1,924 clinical cases during this time. Resistance to fluconazole, amphotericin B, or ≥ 1 echinocandin was detected in 99.5% (1,778/1,787), 39.2% (691/1,761), and 1.8% (33/1,784) of isolates, respectively. Thirteen isolates were resistant to fluconazole, amphotericin B, and ≥ 1 echinocandin. Amphotericin B resistance increased over time (Figure 3).

**Conclusion:**

Detection of *C. auris* cases is increasing in NYC. Focusing infection prevention and control efforts on SNFs might be beneficial. Rising amphotericin B resistance might limit treatment options. AFST results were not available for most isolates, indicating a need for strategies to improve reporting completeness and drug resistance surveillance.

**Disclosures:**

All Authors: No reported disclosures

